# Sulfur and Methane-Oxidizing Microbial Community in a Terrestrial Mud Volcano Revealed by Metagenomics

**DOI:** 10.3390/microorganisms8091333

**Published:** 2020-08-31

**Authors:** Andrey V. Mardanov, Vitaly V. Kadnikov, Alexey V. Beletsky, Nikolai V. Ravin

**Affiliations:** Research Center of Biotechnology of the Russian Academy of Sciences, Institute of Bioengineering, 119071 Moscow, Russia; mardanov@biengi.ac.ru (A.V.M.); vkadnikov@bk.ru (V.V.K.); mortu@yandex.ru (A.V.B.)

**Keywords:** mud volcano, metagenome, sulfur cycle, methane oxidation, ANME archaea, Bathyarchaeota

## Abstract

Mud volcanoes are prominent geological structures where fluids and gases from the deep subsurface are discharged along a fracture network in tectonically active regions. Microbial communities responsible for sulfur and methane cycling and organic transformation in terrestrial mud volcanoes remain poorly characterized. Using a metagenomics approach, we analyzed the microbial community of bubbling fluids retrieved from an active mud volcano in eastern Crimea. The microbial community was dominated by chemolithoautotrophic *Campylobacterota* and *Gammaproteobacteria*, which are capable of sulfur oxidation coupled to aerobic and anaerobic respiration. Methane oxidation could be enabled by aerobic *Methylococcales* bacteria and anaerobic methanotrophic archaea (ANME), while methanogens were nearly absent. The ANME community was dominated by a novel species of Ca. Methanoperedenaceae that lacked nitrate reductase and probably couple methane oxidation to the reduction of metal oxides. Analysis of two Ca. Bathyarchaeota genomes revealed the lack of *mcr* genes and predicted that they could grow on fatty acids, sugars, and proteinaceous substrates performing fermentation. Thermophilic sulfate reducers indigenous to the deep subsurface, *Thermodesulfovibrionales* (*Nitrospirae*) and Ca. Desulforudis (*Firmicutes*), were found in minor amounts. Overall, the results obtained suggest that reduced compounds delivered from the deep subsurface support the development of autotrophic microorganisms using various electron acceptors for respiration.

## 1. Introduction

Mud volcanoes are surface geological features representing the expression of a fracture network that often extends to several kilometers in depth [1]. They are usually found along fracture or fault zones associated with compressional tectonic regions [2,3,4]. Mud volcanoes can be diverse in form but are typically cone-shaped circular structures or pools, from which gaseous mud slurries are discharged, in some cases containing hydrocarbons [2,3]. Methane and CO_2_ appear to constitute the gaseous phase emitted from most mud volcanoes [5]. The contribution of mud volcanoes and seepages to the global greenhouse gas emissions is significant and, according to recent estimates, account for approximately 30% of the natural methane emission [6].

Mud volcanoes attracted the attention of microbiologists because they provide easy access to the deep subsurface biosphere since relatively rapid fluid transport in a fracture network enables limited alteration of fluid and gas until it is discharged to the surface [2]. Microbial communities associated with mud volcanoes located at the seafloor have been extensively studied [7,8,9,10,11,12]. More limited number of studies has focused on microbial communities in terrestrial mud volcanoes [13,14,15,16,17,18,19]. These studies revealed that archaeal communities are mostly composed of methanogens and anaerobic methanotrophs (ANME), while bacterial communities are more diverse and consist of members of various phyla. Among them, sulfate-reducing bacteria, presumably originating from deep subsurface fluids, were found at various terrestrial mud volcano sites [14,16,17,18,20]. Sulfate-reducers can form syntrophic consortia with anaerobic methanotrophic archaea (ANME) performing anaerobic oxidation of methane (AOM) [8,21]. AOM can also be coupled with the reduction of iron, manganese, nitrate, and nitrite [22,23,24]. Consistently, the composition of both archaeal and bacterial communities strongly depends on the concentrations of these potential electron acceptors [17,18].

The mud volcanoes selected for this study are located in the Kerch Peninsula, where the south-eastern end of Crimea extends between the Sea of Azov and the Black Sea. This region, known as the Kerch-Taman mud volcanic province, is one of the most manifested mud volcanism areas located in the Caucasus segment of the Alpine Himalayan belt [3]. This province includes more than 100 active mud volcanoes. Among them, there are episodically active violent mud volcanoes (up to 60 m high), continuously erupting small cone-shaped circular structures, and bubbling mud pools [25]. Until now, microbial communities of the mud volcanoes of the Kerch Peninsula have not been characterized.

The aim of this study was to uncover the composition and functional potential of a mud volcano microbial community. We report data on high-throughput sequencing of 16S rRNA gene amplicons and assembled metagenome of mud fluid. We successfully recovered high-quality metagenome-assembled genomes (MAGs) of the majority of the community members, which enabled accurate metabolic reconstruction of the microbial community, and revealed the primary microbial drivers of methane and sulfur cycling.

## 2. Materials and Methods

### 2.1. Sampling, Field Measurements, Chemical Analyses, and DNA Isolation

The mud volcano sampled in this study is located on the Bulganak mud volcano field, in the Bondarenkovo Village area, near the city of Kerch (45.4261° N 36.4780° E). Mud samples were collected on 18 May 2019, from a ‘bubbling pool’ in the central crater-like structure of the volcano. Samples were collected at a depth of 10–20 cm below the surface of the liquid into sterile 50 mL tubes avoiding gas bubbles. The temperature, pH, and Eh of the recovered fluids were determined on-site using pH-meter HI 8314 (Hanna Instruments, Germany). For chemical analysis, the mud samples were centrifuged at 12,000× *g* for 10 min. The supernatant was filtered through a 0.22-µm sterile filter (Merck Millipore, Darmstadt, Germany) and analyzed by inductively coupled plasma mass spectrometry (ICP-MS) and ion chromatography. Total DNA was extracted from 15 mL of the mud slurry using the DNeasy PowerMax Soil Kit (Qiagen, Hilden, Germany). About 30 ng of DNA was obtained.

### 2.2. 16S rRNA Gene Sequencing and Analysis

PCR amplification of 16S ribosomal RNA gene fragments spanning the V3–V6 variable regions was carried out using the universal primers 341F (5′-CCTAYGGGDBGCWSCAG-3′) and 806R (5′-GGACTACNVGGGTHTCTAAT-3′) [26]. PCR fragments were barcoded using the Nextera XT Index Kit v.2 (Illumina, San Diego, CA, USA). The PCR fragments were purified using Agencourt AMPure beads (Beckman Coulter, Brea, CA, USA) and quantitated using the Qubit dsDNA HS Assay Kit (Invitrogen, Carlsbad, CA, USA). Then, all of the amplicons were pooled together in equimolar amounts and sequenced on the Illumina MiSeq (2 × 300 nt paired-end reads). Paired-end overlapping reads were merged using FLASH v.1.2.11 [27]. The sequences were clustered into operational taxonomic units (OTUs) at 97% identity using the USEARCH program [28]; low quality reads, chimeric sequences, and singletons were removed during clustering by the USEARCH algorithm. To calculate OTU abundances, all reads (including singleton and low-quality reads) were mapped to OTU sequences at 97% global identity threshold by Usearch. The taxonomic identification of OTUs was performed by searches against the SILVA v.132 rRNA sequence database using the VSEARCH sintax algorithm [29]. The OTU sequences are provided in Supplementary Table S1.

### 2.3. Sequencing of Metagenomic DNA, Assembly, and Annotation of MAGs

Metagenomic DNA was sequenced using the Illumina HiSeq2500 platform according to the manufacturer’s instructions (Illumina). The sequencing of a paired-end (2 × 150 bp) TruSeq DNA library generated 59,783,865 read pairs. Adapter removal and trimming of low-quality sequences (Q < 33) were performed using Cutadapt v.1.8.3 [30] and Sickle v.1.33 [31], respectively. Trimmed reads were merged using FLASH v.1.2.11 [27]. The resulting merged and unmerged reads (about 12.8 Gbp in total) were de novo assembled into contigs using metaSPAdes genome assembler v.3.13.0 [32].

Contigs longer than 1500 bp were binned into clusters representing MAGs using CONCOCT v.0.4.1 [33] and MetaBAT v.2.12.1 [34]. The completeness of the MAGs and their possible contamination (redundancy) were estimated using CheckM v.1.05 [35] with lineage-specific marker genes. The assembled MAGs were taxonomically classified using the Genome Taxonomy Database Toolkit (GTDB-Tk) v.0.3.2 and Genome Taxonomy database (GTDB) [36]. The contig to MAG binning scheme was selected manually by comparing the results from MetaBAT and CONCOCT.

Gene search and annotation of MAGs were performed using the National Center for Biotechnology Information (NCBI) Prokaryotic Genome Annotation Pipeline [37] or RAST server 2.0 [38], followed by manual correction of the annotation by comparing the predicted protein sequences with the NCBI databases.

### 2.4. Genome -to-Genome Distance Estimation and Phylogenetic Analysis

The average amino acid identity (AAI) and average nucleotide identity (ANI) between the selected genomes was calculated using scripts from the Enveomics Collection [39].

GTDB-Tk v.0.3.2 was used to find single-copy marker genes in the MAGs and to construct a multiple sequence alignment of concatenated single-copy gene sequences, comprising of those from a given MAG and all species from the GTDB. A portion of the multiple sequence alignment generated in GTDB-Tk was used to construct a phylogenetic tree with PhyML v.3.3 [40] using default parameters. The level of support for internal branches was assessed using the Bayesian test in PhyML.

### 2.5. Nucleotide Sequence Accession Numbers

The raw data generated from 16S rRNA gene sequencing and metagenome sequencing have been deposited in the NCBI Sequence Read Archive (SRA) under the accession numbers SRR11917417 and SRR11917416, respectively (BioProject PRJNA636628). The annotated sequences of MAGs have been deposited in the GenBank database and are accessible via the BioProject PRJNA636628.

## 3. Results and Discussion

### 3.1. Study Site and Fluid Chemistry

**Figure 1:**
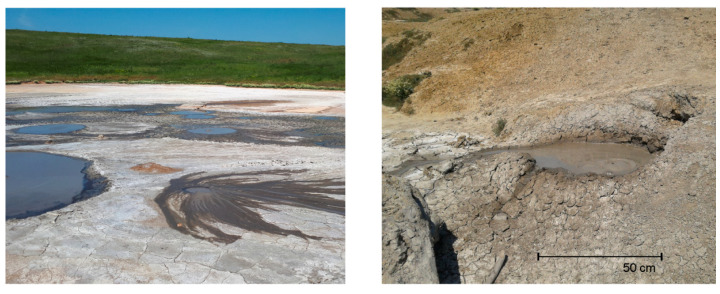
Field images of the Bulganak mud volcano area (**left**) and the studied mud volcano (**right**).

The study site was a mud volcano located on the Bulganak mud volcano field near the city of Kerch. Here, there are more than ten mud volcanoes of two types: relatively large flat mud pools up to 15 m in diameter with liquid mud, and small conical volcanoes with more dense mud. The mud volcano in this study was a cone-shaped structure with a diameter of about 2 m and a height of about 50 cm. The top of the volcano had a central crater-like depression (50–70 cm in diameter), in which bubbling and fluid discharge out of the cone was observed (Figure 1).

The mud temperature was about 16 °C, a value consistent with the origin of the fluid from the subsurface. The fluid had a near neutral pH (about 7.43) and was moderately reduced (Eh = −123 mV). The chemical characteristics of the water extracted from the mud sample are shown in Table 1. The major cation was sodium, followed by boron, calcium, and potassium. Chloride and carbonate were dominant anions, while the concentration of sulfate was rather low (6.1 mg L^−1^). The mass concentration of nitrate, another potential electron acceptor for anaerobic respiration, was nearly twice as high as sulfate.

### 3.2. Microbial Community Structure Revealed by 16S rRNA Profiling

**Figure 2:**
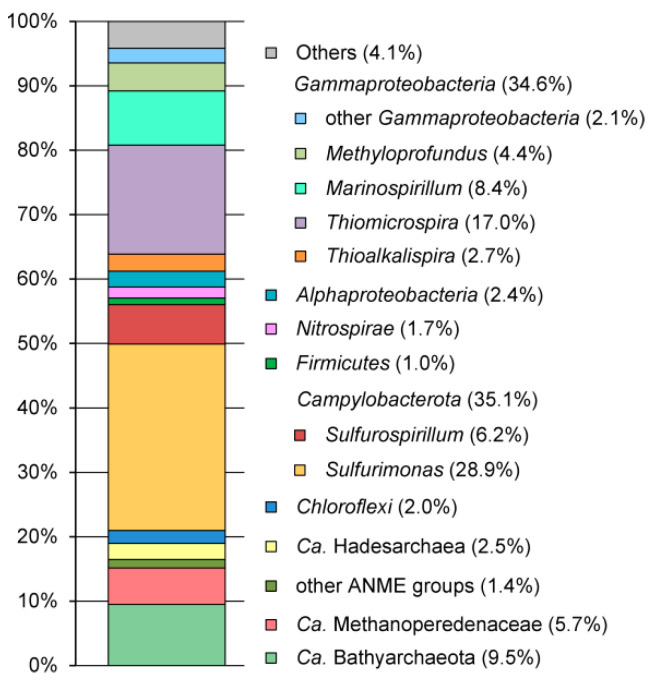
The relative abundance of taxonomic groups of microorganisms according to 16S rRNA gene profiling.

A total of 82,023 high-quality 16S rRNA reads were used to analyze microbial community composition. The pool of reads retrieved from the mud sample was dominated by 16S rRNA gene sequences of bacterial origin (79.7% of the total reads), and archaeal 16S rRNA gene reads accounted for 19.1% of the total reads. About 1.2% of read were assigned to chloroplasts probably appeared as a result of the ingress of allochthonous biomaterial from the surrounding area. The results of the taxonomic classification of the OTUs are shown in Figure 2 and the Supplemental Table S1.

The archaeal population was represented by members of *Candidatus* (*Ca*.) Bathyarchaeota (9.5% of the total reads), *Euryarchaeota* (*Methanomicrobia* at 7.0% and *Halobacteria* at 0.05%), and Ca. Hadesarchaea (2.5%). Nearly all *Euryarchaeota* belonged to lineages known to be involved in anaerobic methane oxidation, namely, Ca. Methanoperedenaceae (5.7%), ANME-3 (0.6%), ANME-2a-2b (0.5%), and ANME-1 (0.3%). Most of Ca. Bathyarchaeota was represented by two OTUs. A GenBank search revealed 16S rRNA sequences closely related (>98% identity) to both of these OTUs in terrestrial and marine mud volcanoes, groundwater, and marine sediments.

The phylum Ca. Hadesarchaea was represented by two OTUs. 16S rRNA gene sequences that were similar to both of them, as revealed by GenBank search, were detected in mud volcanoes worldwide.

The most abundant bacterial lineage, *Campylobacterota* (formerly *Epsilonproteobacteria*), accounted for 35.1% of the community and was represented by the genera *Sulfurimonas* (28.9%) and *Sulfurospirillum* (6.2%). *Sulfurimonas* species are chemolithoautotrophs that are typically found in sulfidic environments such as hydrothermal vents, marine sediments, sulfidic springs, and groundwater [41,42]. They can oxidize various reduced sulfur compounds (some species also hydrogen) using oxygen, nitrate, or nitrite as electron acceptors. Metabolically versatile *Sulfurospirillum* species can use various electron acceptors, including oxygen, nitrate, arsenate, sulfur, and halogenated compounds [43].

The second most frequent lineage was *Gammaproteobacteria* at an abundance of 34.6%, represented by the genera *Thiomicrospira* (17.0%), *Marinospirillum* (8.4%), *Methyloprofundus* (4.4%), *Thioalkalispira* (2.7%), *Marinobacter* (1.0%), *Halomonas* (0.6%), and *Methylomicrobium* (0.2%). Cultured members of the genera *Thiomicrospira* and *Thioalkalispira* are sulfur-oxidizing chemolithoautotrophs [44,45]. The genus *Methyloprofundus* currently consists of a single species, *Methyloprofundus sedimenti*, an obligate methanotroph from oceanic sediments [46]. The heterotrophic part of the community was represented by *Marinospirillum*, *Marinobacter*, and *Halomonas*, which are chemoorganotrophic aerobic halophilic bacteria that are typically found in saline environments, such as soda lakes, marine sediments, and saline soil [47,48,49,50].

About 2.4% of the 16S rRNA reads were assigned to *Alphaproteobacteria* of the family *Rhodobacteraceae* that is mostly comprised of aerobic heterotrophs. *Chloroflexi*, assigned to uncultured lineages of the classes *Anaerolineae* and *Dehalococcoidia*, was present at 2.0%. The phylum *Nitrospirae* (1.7%) was represented by a single OTU that was phylogenetically distant from cultured sulfate-reducing species of *Thermodesulfovibrio*. Among other recognized bacterial lineages, members of *Aminicenantes*, *Actinobacteria*, *Aerophobetes*, *Atribacteria*, *Bacteroidetes*, *Betaproteobacteria*, *Deltaproteobacteria*, *Firmicutes*, and *Spirochaetes* were found in minor amounts (<1% of reads).

### 3.3. Metagenome Sequencing and Assembly of MAGs

To assemble the composite genomes of the most abundant members of the microbial community, we sequenced the metagenome of the mud sample using the Illumina HiSeq2500 platform. A total of 12.8 Gbp of metagenomic sequences were assembled into contigs. These contigs were binned into 35 MAGs with more than 90% completeness and less than 10% contamination (Kmv03 to 05, Kmv11 to 37, and Kmv39 to 43), as estimated based on the presence of a set of single-copy conserved genes by CheckM (Supplementary Table S2).

Taxonomic classification of the MAGs based on the searches against GTDB [36] revealed the same major prokaryotic lineages that were detected by 16S rRNA profiling. To get insights into the metabolic capabilities of the microbial lineages that were most abundant in the microbiome or involved in important biotransformations, we analyzed several of the MAGs in detail.

### 3.4. Sulfur-Oxidizing Bacteria

Six MAGs were assigned to lineages of *Campylobacterota* and *Gammaproteobacteria*, which potentially oxidize reduced sulfur compounds. MAG Kmv11 was classified as a member of *Sulfurimonas*, and the less abundant MAG Kmv12, according in the GTDB taxonomy, represented genus-level division GCA-2733885; both are in the *Sulfurimonadaceae* family. Analysis of the Kmv11 genome revealed a set of genes described in other *Sulfurimonas* genomes that enable oxidation of sulfur compounds [51]. There are two sulfide quinone oxidoreductase (*sqr*) genes, flavocytochrome *c* sulfide dehydrogenase gene, and two gene clusters encoding sulfur oxidation enzymes, *soxXYZAB* and *soxCDYZH*. The presence of *soxXYZAB*, missing in some *Sulfurimonas* species [52], indicates the ability of Kmv11 bacterium to oxidize thiosulfate. The presence of periplasmic group 1a H_2_-uptake [NiFe] hydrogenase suggests that hydrogen also could be used as an electron donor. The Kmv11 genome encodes NADH-ubiquinone oxidoreductase, succinate dehydrogenase, cytochrome *bc* complex, and terminal reductases for aerobic and anaerobic respiration. The presence of Nap-type periplasmic nitrate reductase, cytochrome *cd1*-dependent nitrite reductase, nitrous oxide reductase, and nitric-oxide reductase indicates the ability of *Sulfurimonas* Kmv11 bacterium to perform complete denitrification. The presence of cytochrome *c* oxidase of the *cbb_3_*-type known to have a high affinity for oxygen [53], suggest that this bacterium could be capable of aerobic respiration under microaerophilic conditions.

Two MAGs were assigned to the family *Sulfurospirillaceae* proposed in the GTDB taxonomy. One of them, Kmv13, belonged to the genus *Sulfurospirillum*, while a less abundant MAG Kmv14 was assigned to the genus-level division UBA1877. Analysis of the Kmv13 genome revealed that contrary to Kmv11 *Sulfurimonas*, this bacterium lacked a complete sulfur-oxidation pathway since only a sulphide quinone oxidoreductase and a *soxCDYZH* cluster are present. Genome analysis indicated the possibility that various electron acceptors are used for respiration. The Kmv13 genome contained *napAGHBFLD* genes of periplasmic nitrate reductase, a nitrous oxide reductase gene cluster similar to the *nos* cluster in *Sulfurospirillum arsenophilum* [54], an ammonia-forming cytochrome *c* nitrite reductase *nrfAH*, a respiratory arsenate reductase, and a Psr/Psh family molybdopterin reductase that could be responsible for reduction of sulfur compounds, such as thiosulfate or polysulfide. In addition, the *Sulfurospirillum* Kmv13 genome contains a five gene cluster encoding three multiheme *c*-type cytochrome (MHC), an iron-sulfur electron-transfer PsrB-like protein, and a PsrC-like membrane anchor. This gene cluster is absent in other sequenced genomes of *Sulfurospirillum* sp. The presence of an N-terminal secretion signal in two *c*-type cytochromes indicates their extracytoplasmic localization. Such *c*-type multiheme cytochromes play a key role in electron transfer to the extracellular electron acceptor in iron-reducing *Shewanella* and *Geobacter* sp. [55]. The reductive dehalogenase gene cluster, enabling organohalide respiration in *Sulfurospirillum multivorans* [56], and the *nor* operon coding for nitric oxide reductase were absent. Similar to Kmv11 *Sulfurimonas,* the Kmv13 bacterium contained the *cbb3*-type cytochrome *c* oxidase and likely grows by oxygen respiration under low oxygen concentration. The *Sulfurospirillum* Kmv13 genome encoded a complete tricarboxylic acid (TCA) cycle, and the presence of ATP citrate lyase and fumarate reductase suggests the possibility of its operation in reverse, for CO_2_ fixation. Like other *Sulfurospirillum* species, the Kmv13 bacterium has enzymes enabling the use of lactate and acetate as carbon sources, as well as a respiratory H_2_-uptake [NiFe] hydrogenase.

The genus *Thiomicrospira* of *Gammaproteobacteria* was represented by MAG Kmv17. Genes encoding all of the components of a Sox system, namely *soxXYZABCD* and two sulfide quinone oxidoreductase genes, were identified. Like in *Thiomicrospira crunogena* [57], *sox* genes were not organized in a single cluster but were located in different regions of the Kmv17 genome: *soxXYZA*, *soxB*, and *soxCD*. The [NiFe] hydrogenases enabling hydrogen uptake were absent, suggesting that *Thiomicrospira* Kmv17 cannot use it as an electron donor along with sulfur compounds, unlike some other *Thiomicrospira* species [58]. A complete aerobic respiratory chain was encoded, with a terminal *cbb_3_*-type cytochrome *c* oxidase. Contrary to *Sulfurimonas* and *Sulfurospirillum*, Kmv17 lacked any recognizable reductases for anaerobic respiration and probably only grows aerobically. Like other *Thiomicrospira* species, Kmv17 could fix CO_2_ via the Calvin–Benson–Bassham cycle. Kmv17 had a complete TCA cycle, but it most likely only operates in the oxidative direction, as evidenced by the absence of citrate lyase gene. Overall, *Thiomicrospira* Kmv17 bacterium seems to be a highly specialized chemolithoautotroph devoted to oxidation of sulfur compounds under aerobic conditions.

MAG Kmv18 was classified as a novel genus-level member of GTDB family *Thiohalomonadaceae* and likely corresponded to the *Thioalkalispira*-related OTU, identified by 16S rRNA gene profiling. The sulfur-oxidizing inventory of genes includes sulfide quinone oxidoreductase, *soxXYZAB* oxidase, and *soeABC* sulfite dehydrogenase. Like Kmv17, this genome lacked uptake hydrogenases, terminal reductases for anaerobic respiration, but encoded the Calvin–Benson–Bassham cycle for CO_2_ fixation, and a single terminal cytochrome *c* oxidase of the *cbb_3_*-type.

### 3.5. Sulfate-Reducing Bacteria

Two likely sulfate-reducing species were identified in the metagenome. MAG Kmv42 was assigned to the GTDB order *Thermodesulfovibrionales*, whose cultured members, *Thermodesulfovibrio* species, can perform sulfate reduction [59]. This bacterium is phylogenetically distant from cultured species and was classified in the candidate GTDB family SM23-35. The second MAG (Kmv41) was phylogenetically related to Ca. Desulforudis audaxviator (72.5% AAI, 77.1% ANI), an enigmatic chemolithoautotrophic sulfate-reducing Firmicutes, thriving in terrestrial deep subsurface environments [60,61], and to Ca. Desulfopertinax cowenii (77.2% AAI, 78.8% AN) from basalt-hosted fluids of the deep subseafloor [62]. The absence of the 16S RNA gene in the Kmv41 MAG did not allow to define more precisely its phylogenetic relationship with these candidate species. Evaluation of the Kmv41 and Kmv42 genomes revealed the presence of a complete dissimilatory sulfate-reduction pathway, including sulfate adenyltransferase, adenosine-5′-phosphate reductase, dissimilatory sulfite reductase, and associated redox complexes Qmo and DsrMK (in Kmv41)/DsrMKJOP (in Kmv42). The Ca. Desulforudis Kmv41 genome contains the complete genetic complement for autonomous chemolithoautotrophic growth, including respiratory hydrogenases supplying electron acceptors for sulfate reduction, the Wood–Ljungdahl pathway, and nitrogenase. The *Thermodesulfovibrionales* MAG Kmv42 also encoded respiratory hydrogenases and the Wood–Ljungdahl pathway for carbon fixation, but it lacked a nitrogenase. The Kmv42 bacterium is metabolically more versatile and can use proteinaceous substrates and carbohydrates for growth, as evidenced by the presence of hydrolytic enzymes and transporters for the uptake of sugars, peptides and amino acids.

### 3.6. Bacteria and Archaea Involved in the Methane Cycle

**Figure 3:**
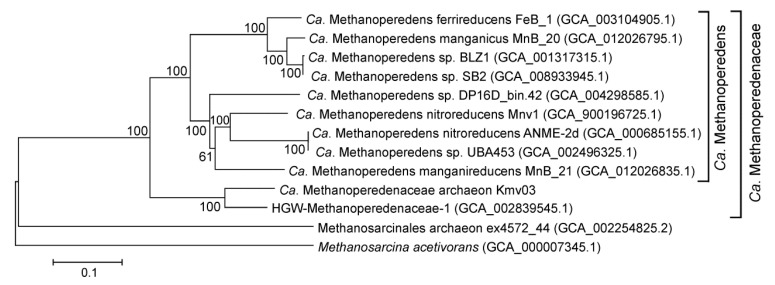
Phylogenetic placement of the Ca. Methanoperedeaceae archaeon Kmv03. The genome tree was inferred using the maximum-likelihood method with a concatenated set of 122 archaeal-specific marker genes. GenBank assembly accession numbers are shown in parentheses after the genome names. The genome of *Methanosarcina acetivorans* was used to root the tree.

Three archaeal MAGs belonged to known ANME lineages. The most abundant MAG, Kmv03, was assigned to the family Ca. Methanoperedenaceae, also known as ANME-2d group. These archaea could couple anaerobic oxidation of methane to the reduction of nitrate (*Ca*. Methanoperedens nitroreducens, [63]), Fe^3+^ (*Ca*. M. ferrireducens, [64]) or Mn^4+^ (*Ca*. M. manganicus and Ca. M. manganireducens [65]). The Kmv03 archaeon is most closely related (83% AAI) to Ca. Methanoperedenaceae archaeon HGW-Methanoperedenaceae-1, recovered from the metagenome of a groundwater sample collected at depths of 140–250 m in Japan [66]. Together these two MAGs formed a distinct genus-level lineage in the family Ca. Methanoperedenaceae, which was second to the previously described Ca. Methanoperedens [63] (Figure 3).

The Kmv03 genome encoded a complete reverse methanogenesis pathway and related energy converting enzymes, including the cytoplasmic and membrane-bound CoB-CoM heterodisulfide reductase, F_420_H_2_ dehydrogenase, sodium-translocating methyltransferase complex (Mtr), and V-type ATPase. The nitrate reductase complex, responsible for nitrate reduction coupled to AOM in Ca. M. nitroreducens [67], was not encoded in the Kmv03 genome. Therefore, Kmv03 likely use electron acceptors other than nitrate. The ability of Ca. M. ferrireducens, Ca. M. manganicus, and Ca. M. manganireducens to reduce insoluble Fe(III) or Mn(IV) oxides was attributed to MHCs [65,68]. Analysis of the Kmv03 genome revealed 18 MHCs containing up to 22 hemes, of which 18 cytochromes were predicted to contain an N-terminal secretion signal. In particular, we identified a gene cluster containing two pentaheme *c*-type cytochromes, cytochrome *c* with a single heme, two membrane proteins, and two membrane-integral cytochrome *b* proteins. All three cytochrome *c* proteins, besides the N-terminal secretion sequence, contained C-terminal transmembrane helices, indicating they were localized on the external side of the cell membrane. In addition to MHCs, archaeal flagellar-like conductive structures were proposed to be involved in the long-distance transfer of electrons between ANME archaea and their sulfate-reducing partners [69] or metal oxides [65]. Kmv03 contained genes involved in the formation of the archaellum, including two *flaB* genes encoding for archaellin. Since MHCs and archaella in different ANME lineages could be involved in the electron transfer to their syntrophic partners or insoluble electron acceptors [65,68,69], we could not predict which function was relevant for Ca. Methanoperedenaceae Kmv03 archaeon. However, the abundance of potential metal-reducing bacteria carrying MHCs in the microbial community and the minor fraction of sulfate reducers favors the hypothesis that metal oxides are used as electron acceptors for AOM.

Two other, less abundant ANME lineages, ANME-2a-2b of the *Methanosarcinales* and ANME-1, were represented by MAGs Kmv04 and Kmv05, respectively. Both of these ANME groups are known to be associated with sulfate-reducing *Deltaproteobacteria* [9,70], but the latter were not identified by 16S rRNA profiling or among the MAGs.

Most of bacterial methanotrophs were represented by MAG Kmv24, assigned to the genus *Methyloprofundus*, with 72.2% AAI to *Methyloprofundus sedimenti*. Analysis of the Kmv24 genome revealed the presence of both soluble and particulate methane monooxygenases, methanol dehydrogenase, and downstream enzymes required for the metabolism of formaldehyde. Three cytochrome *c* oxidases were identified, while enzymes for dissimilatory reduction of nitrate and other nitrogen compounds were absent. All of these traits were consistent with methanotrophic lifestyle. Two other MAGs, Kmv25 and Kmv26, represented well-characterized groups of aerobic methanotrophic bacteria and were assigned to the genera *Methylomicrobium* and *Methylophaga*, respectively.

### 3.7. Iron-Reducing Deltaproteobacteria

*Deltaproteobacteria*, according to the 16S rRNA profiling, only accounted for 0.34% of the community and belonged to the order *Desulfuromonadales*. Nevertheless, two high-quality MAGs, Kmv15 and Kmv16, were obtained and assigned to *Desulfuromonadales*. Although many members of this order are sulfate-reducers, analysis of the Kmv15 genome revealed the absence of a dissimilatory sulfate-reduction pathway. The hallmark of this genome is the presence of a complete pathway for beta-oxidation of fatty acids, including fatty-acid-CoA ligases, two copies of *fadN-fadA-fadE* operon, propionyl-CoA carboxylase, methylmalonyl-CoA mutase, and methylmalonyl-CoA epimerase. Oxidation of lactate to pyruvate is probably enabled by a multi-subunit lactate dehydrogenase belonging to the CCG family, which were found in the genomes of various *Deltaproteobacteria*, particularly in *Desulfovibrio vulgaris* [71]. Fatty acids and lactate are most likely the main substrates, since no genes encoding extracellular glycoside hydrolases and proteases were found.

Besides fermentation, Kmv15 bacterium is capable of anaerobic respiration. Its genome encoded a molybdopterin family respiratory arsenate reductase, cytochrome *c* nitrite reductase, and more than a dozen of MHCs with N-terminal secretion signals. These cytochromes could be necessary to contact the insoluble electron acceptors and probably enable dissimilatory Fe(III) reduction, as reported in *Geobacter* species [55]. Adhesion to Fe(III) minerals could also be facilitated by the type IV pili encoded by the Kmv15 genome. Hydrogen could be used as an electron donor, as indicated by the presence of two group 1 respiratory H_2_-uptake [NiFe] hydrogenases. Three membrane-linked complexes, NADH-ubiquinone oxidoreductase, an electron transport Rnf complex, and Na^+^-translocating NADH-quinone reductase, can generate a transmembrane ion gradient, which can be employed by F_0_F_1_ ATP synthase for ATP production. Overall, the genome analysis revealed that Kmv15 is an organotrophic bacterium specialized in utilizing fatty acids to perform fermentation and anaerobic respiration with arsenate, nitrite, and Fe(III).

The second MAG, Kmv16, was assigned in the GTDB taxonomy to another candidate family of *Desulfuromonadales*. Like Kmv15, the Kmv16 genome lacked a dissimilatory sulfate reduction pathway, and contained genes for beta-oxidation of fatty acids, respiratory arsenate reductase, multiheme cytochromes *c*, NADH-ubiquinone oxidoreductase, Rnf complex, sodium-translocating NADH-quinone reductase, group 1 respiratory [NiFe] hydrogenases, and L-lactate dehydrogenase. The cytochrome *c* nitrite reductase was not found, but respiratory nitrate reductase was present. Contrary to Kmv15, Kmv15 bacterium possesses an aerobic respiratory chain with two cytochrome *c* oxidases: *caa3* and *cbb3* types. These oxidases vary in their affinities for oxygen and could enable respiration under fluctuating oxygen concentrations.

### 3.8. Bathyarchaeota

The archaea of the uncultured candidate phylum *Bathyarchaeota* (recognized as a class of the phylum *Crenarchaeota* in the GTDB taxonomy) accounted for almost 10% of all the 16S rRNA gene sequences. Analysis of *Bathyarchaeota* genomes revealed that these organisms are organoheterotrophs that possibly utilize various proteinaceous substrates and polysaccharides of plant origin [72]. Their genomes encoded the Wood-Ljungdahl pathway of autotrophic carbon fixation. Depending on the environmental conditions, *Bathyarchaeota* can use this pathway for acetogenesis or in reverse, using acetate to form H_2_ and CO_2_ [73]. Genomes of some members of *Bathyarchaeota* contain Mcr-like genes, and it was suggested that they perform methyl-dependent hydrogenotrophic methanogenesis [74]. However, it was later proposed that these genes are probably involved in the oxidation of short-chain alkanes rather than methanogenesis [75]. Assembly of two *Bathyarchaeota* MAGs with an estimated completeness of 86% (Kmv01) and 89% (Kmv02) enabled to get insights into the metabolic potential of these archaea.

According to GTDB taxonomy, these MAGs were assigned to families UBA233 and BA1 of the order B26-1. Notably, two potential methanogenic members of the *Bathyarchaeota*, Ca. Bathyarchaeota archaeon BA1 and Ca. Bathyarchaeota archaeon BA2, belonging to the BA1 family, were found in the formation waters from a coalbed methane well [74]. Kmv02 and BA2 archaeons likely belong to the same genus sharing a 74% AAI. The genome size of Kmv02 (1,674,670 bp) was similar to that of BA1 (1,931,714 bp) and BA2 (1,455,689 bp). Meanwhile, Kmv01 had a smaller genome (1,046,189 bp). Considering the close phylogenetic relatedness of Kmv02 and BA1/BA2 archaea, the absence of methyl-coenzyme M reductase genes in the Kmv02 genome was rather unexpected. The Mtr complex was also missing. The second MAG, Kmv01, also lacked all *mcr* and *mtr* genes. Considering that these MAGs were estimated to be 86% and 89% complete, the probability that *mcr* and *mtr* genes were missed in the assemblies by chance is unlikely; therefore, both bathyarchaeons are probably not involved in methanogenesis or short-chain alkane oxidation dependent on Mcr-like proteins.

The search for carbohydrate-active enzymes in the genome of *Bathyarchaeota* Kmv02 revealed GH57 family alpha-amylase and alpha-mannosidase, and GH1 family beta-galactosidase/beta-glucosidase, all of which lacked N-terminal signal peptides, and several ABC-type sugar transporters. The genome also encoded ABC-type peptide transporters, peptidases, aminotransferases, and 2-oxoacid ferredoxin oxidoreductases, enabling fermentation of proteinaceous substrates. The genome encoded a near-complete Embden–Meyerhof glycolysis pathway and enzymes required for gluconeogenesis (i.e., fructose-1,6-bisphosphatase and phosphoenolpyruvate synthase). In addition, the beta-oxidation pathway for fatty acids utilization was present, including long-chain-fatty-acid-CoA ligases, enoyl-CoA hydratases, 3-ketoacyl-CoA thiolases, and acyl-CoA dehydrogenases. Therefore, the Kmv02 archaeon might utilize a wide range of organic substrates, but it cannot degrade complex polymers.

Pyruvate produced in fermentation pathways could be converted to formate and acetyl-CoA by pyruvate formate-lyase, or oxidized by pyruvate:ferredoxin oxidoreductase. The acetyl-CoA produced may either enter the Wood–Ljungdahl pathway, or it could be oxidized to acetate with ATP production by acetyl-CoA synthetase. Four [NiFe] hydrogenases were identified in the *Bathyarchaeota* Kmv02 genome. The first is group 3c heterodisulfide reductase-linked complex (MvhADG-HdrABC) that could bifurcate electrons from H_2_ to heterodisulfide (CoM-S-S-CoB) and ferredoxin [76]. The second is cytoplasmic group 3b cofactor-coupled bidirectional sulfhydrogenase. Together these hydrogenases could re-oxidize reduced cofactors generated in fermentation reactions. In addition, there are two group 4g H_2_-evolving membrane-linked hydrogenases that may form respiratory complexes that couple ferredoxin oxidation with proton reduction and translocate protons across the membrane. Usually, the generated proton motive force is coupled to ATP synthesis, but the Kmv02 genome lacked genes encoding membrane-bound ATP synthases. The ATP synthases were not found also in the genome of Kmv01. The transmembrane proton gradient is probably only utilized for transport purposes. Consistently with fermentative heterotrophic lifestyle, both *Bathyrchaeota* genomes lacked genes for aerobic and anaerobic respiration.

### 3.9. An overview of Microbial Processes in the Mud Volcano

**Figure 4:**
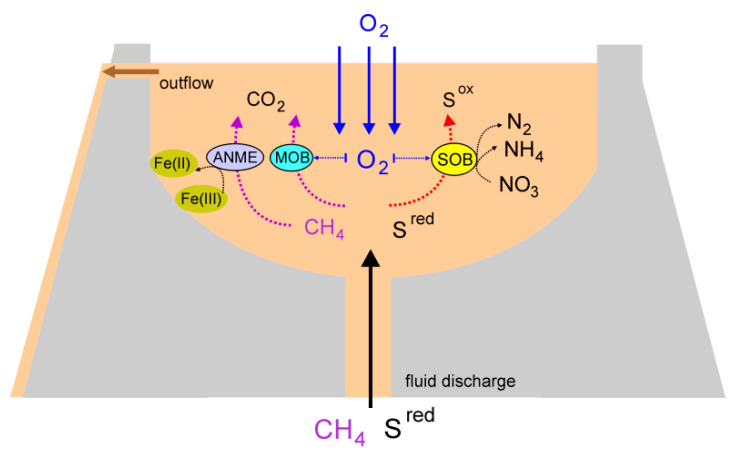
Microbial processes related to sulfur and methane cycling in the mud volcano. SOB, bacteria performing aerobic and/or anaerobic oxidation of sulfur compounds; MOB, aerobic methanotrophic bacteria.

Fluids and gases discharged from mud volcanoes are thought to originate from deep horizons and provide an abundant source of electron donors for microbial growth. Reaching the surface, the reduced mud fluids are exposed to atmospheric oxygen, which provides conditions for the proliferation of microorganisms with a wide range of oxygen requirements and metabolic capabilities (Figure 4). This facilitates the energy and material exchange at the oxic–anoxic interface [77]. In some mud volcanoes, such interactions could lead to geochemical and metabolic stratification forming the upper sulfate-rich oxic and lower methane-rich anoxic zones, harboring distinct bacterial and archaeal communities [14]. For example, in a methane-rich mud volcano in southwestern Taiwan, oxygen penetration was limited to the upper 4 mm layer of the fluids and counteracted by the oxidation of sulfide, methane, and organic matter [16]. However, in the mud volcano we studied, the active release of gases and bubbling created conditions for mud mixing in the crater, at least at the depth from which the samples have been acquired (10–20 cm).

Metagenomic analysis of the mud sample revealed that the microbial community was dominated by chemolithoautotrophic sulfur-oxidizing *Campylobacterota* and *Gammaproteobacteria*. Altogether the sulfur-oxidizing bacteria accounted for about 55% of the 16S rRNA genes sequences. On the contrary, sulfate reducers were found in minor amounts and probably do not play an important role in the sulfur cycle in the mud pool. Members of the *Campylobacterota* appeared to be metabolically versatile being capable of using both sulfur compounds and molecular hydrogen as electron donors. In addition to aerobic respiration under microaerophilic conditions, they had the genetic capacity for anaerobic growth using various electron acceptors, including nitrate, nitrite, nitrogen oxides, arsenate, and sulfur compounds. The presence of about 200 µM of nitrate should enable the growth of these bacteria in the anoxic zones. The sulfur-oxidizing members of the *Gammaproteobacteria* were predicted to only oxidize sulfur compounds under aerobic conditions.

Availability of methane among the emitted gases and the presence of various electron acceptors could support the growth of methanotrophs (Figure 4). Consistently, we found aerobic methanotrophs, gamma-proteobacteria of the order *Methylococcales*, and ANME archaea. The latter can inhabit local anaerobic niches, for example, ones associated with clay particles and the walls of the crater, or be delivered from deeper anaerobic zones with the discharged fluids. ANME archaea are typical inhabitants of microbial communities associated with mud volcanoes [18]. Molecular studies of a mud volcano in eastern Taiwan revealed that the ANME-2a/2b group was most abundant among archaea, and it was suggested that they form syntrophic consortia with *Desulfuromonadales* to facilitate electron transport during AOM [18]. Both of these two lineages were identified in our study, but only in minor amounts. The ANME community was dominated by a novel species of an ANME-2d group, Ca. Methanoperedenaceae Kmv03. Members of the Ca. Methanoperedenaceae can couple AOM to the reduction of nitrate, Fe(III), or Mn(IV) [63,64,65] without a syntrophic partner. Although nitrate was available in the mud breccia, Ca. Methanoperedenaceae Kmv03 archaeon lacked nitrate reductase and most likely relies on the reduction of metal oxides. The reduction of insoluble metal minerals could be an essential process in the mud volcano community. The genetic capacity for this process has been identified in the genomes of various other community members, such as *Sulfurospirillum*, and two *Desulfuromonadales* species.

Organic matter produced by autotrophic sulfur-oxidizing bacteria and methanotrophs could support the heterotrophic part of the community comprising of both aerobic organotrophs (i.e., *Marinospirillum, Marinobacter*, *Halomonas*, *Rhodobacteraceae* sp.) and fermentative organisms. According to the 16S rRNA profiling, *Bathyarchaeota* was the most abundant among the second group. Like ANME archaea, *Bathyarchaeota* probably occupies local anaerobic environments.

The fluids discharged from mud volcanoes are expected to deliver microorganisms from the deep subsurface to the surface where they became mixed with those actively proliferating in the mud breccia. Two groups of presumably thermophilic sulfate reducers indigenous to the deep subsurface have been detected: members of the order *Thermodesulfovibrionales* (*Nitrospirae*) and the genus Ca. Desulforudis (*Firmicutes*). *Thermodesulfovibrio* species are widespread in different hydrothermal habitats, including the deep thermal aquifers [78], while Ca. Desulforudis have been detected exclusively in the deep subsurface [60,61,62]. Interestingly, other typical members of the microbial communities of the deep subsurface ecosystems, thermophilic methanogenic archaea [79], were not found. This might be explained by the source of the fluids. Probably, the fluids originate from the sulfate-rich subsurface site with predominantly sulfate-reducing microbial population such as South African deep subsurface site where Ca. Desulforudis audaxviator formed a single-species ecosystem [60]. Sulfide produced by sulfate reducers in the deep subsurface is transferred to the surface when fluid is discharged and supports the development of the sulfur-oxidizing microbial community in the mud breccia.

Overall the results obtained suggest that reduced sulfur compounds and methane delivered from the deep subsurface support the development of chemolithoautotrophic microbial community using various electron acceptors for respiration.

## Figures and Tables

**Table 1:** Physical and chemical characteristics of the water extracted from the mud sample.

Parameter (Unit)	Value	Parameter (Unit)	Value
Temperature (°C)	16	Sr (mg L^−1^)	2.4
pH	7.43	Li (mg L^−1^)	1.9
Eh, mV	−123	Ba (mg L^−1^)	1.0
Na (mg L^−1^)	4165	Fe (mg L^−1^)	0.4
B (mg L^−1^)	337	As (mg L^−1^)	0.06
K (mg L^−1^)	26	Cl^−^ (mg L^−1^)	1865
Ca (mg L^−1^)	58	NO_3_^−^ (mg L^−1^)	12.0
Mg (mg L^−1^)	23	PO_4_^3−^ (mg L^−1^)	8.3
Si (mg L^−1^)	17	SO_4_^2−^ (mg L^−1^)	6.1

## Supplementary Materials

The following are available online at https://www.mdpi.com/2076-2607/8/9/1333/s1, Table S1: Relative abundance and taxonomic classification of OTUs; Table S2: Main characteristics of MAGs obtained in this study.
